# Structure-function relationships governing activity and stability of a DNA alkylation damage repair thermostable protein

**DOI:** 10.1093/nar/gkv774

**Published:** 2015-10-10

**Authors:** Giuseppe Perugino, Riccardo Miggiano, Mario Serpe, Antonella Vettone, Anna Valenti, Samarpita Lahiri, Franca Rossi, Mosè Rossi, Menico Rizzi, Maria Ciaramella

**Affiliations:** 1 Institute of Biosciences and Bioresources, National Research Council of Italy, Via P. Castellino 111, 80125 Naples, Italy; 2 DiSF-Dipartimento di Scienze del Farmaco, University of Piemonte Orientale ‘A. Avogadro’, Via Bovio 6, 28100 Novara, Italy

## Abstract

Alkylated DNA-protein alkyltransferases repair alkylated DNA bases, which are among the most common DNA lesions, and are evolutionary conserved, from prokaryotes to higher eukaryotes. The human ortholog, hAGT, is involved in resistance to alkylating chemotherapy drugs. We report here on the alkylated DNA-protein alkyltransferase, *Ss*OGT, from an archaeal species living at high temperature, a condition that enhances the harmful effect of DNA alkylation. The exceptionally high stability of *Ss*OGT gave us the unique opportunity to perform structural and biochemical analysis of a protein of this class in its post-reaction form. This analysis, along with those performed on *Ss*OGT in its ligand-free and DNA-bound forms, provides insights in the structure-function relationships of the protein before, during and after DNA repair, suggesting a molecular basis for DNA recognition, catalytic activity and protein post-reaction fate, and giving hints on the mechanism of alkylation-induced inactivation of this class of proteins.

## INTRODUCTION

Alkylated DNA-protein alkyltransferases (AGTs, MGMTs or OGTs, EC 2.1.1.63) are conserved proteins that repair alkylation damage in DNA, manly at position *O^6^* of guanines. They use a peculiar single-step mechanism in which the direct repair of the alkylated base is coupled with irreversible alkylation of the catalytic cysteine in the protein active site. The trans-alkylated protein is permanently inactivated and prone to degradation both *in vivo* and *in vitro* (1–4). Most knowledge these proteins comes from classic studies on Ada-C and OGT of *Escherichia coli*, as well as the human hAGT (1–4). In light of the observation that hAGT over-expression in tumor cells is frequently associated with resistance to alkylating agents, hAGT has received attention as a potential target for the development of treatments to be integrated into current chemotherapy protocols based on such drugs (5).

The current understanding of the AGTs molecular mechanism responsible for alkylated DNA recognition and repair is mainly based on the 3D structures of hAGT and its complex with double-stranded (ds) DNA molecules (6–9). The protein contacts the DNA minor groove through the helix-turn-helix (HTH) motif of its C-terminal domain. By adopting an extra-helical conformation stabilized by an ‘arginine finger’ (R128) of the HTH, the damaged base is deeply inserted in the protein active site, and the alkyl moiety is then transferred to the catalytic cysteine (C145). Data on structure and properties of alkylated AGTs are limited, because alkylation greatly destabilizes their folding; as for 3D structures, the only available are those of the methylated (hAGT^m^) and benzylated (hAGT^b^), which were obtained from native hAGT crystals flash frozen upon soaking in solutions containing *O^6^*-methyl- (*O^6^*-MG) and *O^6^*-benzyl-guanine (*O^6^*-BG), respectively (7). The hAGT^m^ and hAGT^b^ structures showed that C145 alkylation induces subtle conformational changes at the active site; however, these structures might not reflect the physiological conformation of the alkylated hAGT, since in the crystalline state the protein could not accurately display the conformation adopted in solution. Indeed, it is likely that protein movements are restricted in crystals, and larger rearrangements may lead to crystal decay (7).

Alkylation-induced instability of AGTs is interesting from both a biological and mechanistic point of view. AGTs degradation is an important process in the organisms’ response to alkylation damage; in human cells, hAGT physically associates and undergoes repair-mediated degradation with the BRCA2/FancD1 protein (10). Germline mutations of Brca2 are associated with cancer prone syndromes and Fanconi anemia, and absence of a functional BRCA2 protein induces increased cell sensitivity to DNA crosslinking agents (10); thus, hAGT activity and degradation might also affect other DNA repair pathways. The relation between active site modification and protein unfolding/degradation has been difficult to study due to the instability of alkylated AGT forms. *In viv*o, conformational changes might expose residues, which are target for ubiquitination, thus triggering the protein degradation; on the other hand, alkylated hAGT is also intrinsically unstable *in vitro* (7,11). It has been suggested that alkylation-induced conformation modifications induce distortion of the DNA binding surface, facilitating the dissociation of alkylated hAGT from DNA, while destabilizing the protein native fold (7,12,13). Recently, two glycine residues (G131 and G132) were proposed to be implicated in the balance between stability and instability of hAGT, stabilizing the protein in the native form and triggering its destabilization upon alkylation, through a still unclear mechanism (14).

AGTs are present in organisms from the three living domains (Eucarya, Bacteria, Archaea). In thermophilic bacteria and archaea, living at >80°C, alkylation damage is a serious harm since alkylated bases are unstable at high temperature and induce DNA ruptures (15,16). We have previously reported on OGT from the archaeon *Sulfolobus solfataricus* (*Ss*OGT), a protein with outstanding stability at high temperature, which, upon alkylation, becomes unstable and undergoes degradation *in vivo*, suggesting that it follows the same fate as hAGT (17,18).

We present here a biochemical, structural and mutational analysis of *Ss*OGT. The crystal structure of the ligand-free protein and its complex with an *O^6^*-MG containing dsDNA revealed overall similarities with the corresponding structures of hAGT, but also peculiarities, which were found to have functional significance. Moreover, in contrast to the corresponding hAGT^m^, the methylated form of *Ss*OGT (*Ss*OGT^m^) was soluble and relatively stable, thus allowing in-deep analysis of the protein in its post-reaction form. Structural and biochemical analysis of *Ss*OGT^m^, as well as of a mutant mimicking the presence of a bulkier adduct in the active site (C119L), suggested a possible mechanism of alkylation-induced *Ss*OGT unfolding and degradation. Based on our data, we suggest a general model for the mechanism of post-reaction AGTs destabilization.

## MATERIALS AND METHODS

### DNA mutagenesis and protein purification

Site-directed mutants were obtained by using the oligonucleotide ‘mut’ and ‘rev’ pairs (Supplementary Table S1) and the GeneTailor™ Site-Directed Mutagenesis System (Invitrogen); the template was the *S. solfataricus* ogt gene cloned in the pQE31™ vector (17). N-terminally His-tagged proteins were expressed in the *E. coli* ABLE-C strain and purified as described (17).

### Fluorescent assays for *O^6^*-alkyl-guanine alkyltransferase activity

*Ss*OGT activity was determined by a fluorescent assay, by using the SNAP-Vista Green™ (BG-VG) substrate under the standard reaction conditions as previously reported (17,19). After SDS-PAGE, fluorescent protein bands were visualized by gel imaging using the VersaDoc 4000™ system (Bio-Rad). The fluorescence intensity of each band was corrected for the amount of protein loaded by Coomassie Blue staining. Determination of pseudo-first-order reaction rate values was carried out under standard conditions, taking protein aliquots at different times. Second-order rate constants were then obtained by dividing values by the substrate concentration (17,20).

### Direct DNA repair activity

The efficiency of repair of ds oligonucleotides containing a single *O^6^*-MG was determined by incubation of fixed amounts of protein (5 μM) for different timespans at 25°C in the presence of increasing concentrations of the ds-Fwd^m26^ oligonucleotide. Then, 10 μM of BG-VG was added, incubation was prolonged for 2 h and samples analyzed by SDS–PAGE. Gel imaging and Coomassie correction was performed as described above.

### Competition assay with non-fluorescent *O^6^*-methyl-guanine containing DNA

The efficiency of repair of ds oligonucleotides containing a single *O^6^*-MG was determined by using the half maximal inhibitory concentration (IC_50_) method, where IC_50_ is the concentration of methylated DNA needed to reduce by 50% the fluorescence intensity of the *Ss*OGT band in reactions containing fixed concentrations of the fluorescent BG-VG molecule (Supplementary Figure S1A). Reactions were incubated at fixed temperatures with increasing concentrations (0–10 μM) of the appropriate methylated oligonucleotide. Gel imaging and Coomassie correction were performed as described above; corrected data of fluorescence intensity were fitted with the IC_50_ equation. For competitive inhibition, the affinity of the inhibitor (K_DNA_) is related to the IC_50_ value by the adapted Cheng-Prusoff Equation (1) (21):

(1) \begin{equation*} {\rm K}_{{\rm DNA}} = {\rm IC_{50}}/[1+([{\rm VG}]/{\rm K_{VG}})] \end{equation*}

where K_DNA_ is the binding affinity of the inhibitor, IC_50_ is the functional strength of the inhibitor, [VG] and K_VG_ are the VG concentration and the concentration of the BG-VG substrate at which enzyme activity is half maximal, respectively (17).

### Electrophoretic mobility shift assay

A tetramethylrhodamine labeled dsDNA probe was prepared by annealing the oligonucleotides A^+^ and D^−^, as reported (Supplementary Table S1; 17). Different amounts of protein (0.0–25.0 μM) were incubated at 37°C for 10 min with the probe (0.2 μM), in a total volume of 10.0 μl, as described (17). After loading the sample on an 8% polyacrylamide native gel run in 1X TBE (90.0 mM Tris-HCl, 90.0 mM boric acid, 2.0 mM EDTA, pH 8.3), signals were measured by gel imaging, using a green LED/605 bandpass filter as excitation/emission parameters, respectively. For DNA binding assay with unlabeled dsDNA oligonucleotides containing *O^6^*-MG, samples were prepared as described above, except that, after electrophoresis, gels were stained with ethidium bromide (10 mg/ml) for 15 min at RT.

### Protein stability assays

Thermal stability was determined by two different methods. (i) temperature-induced aggregation: 30 μl aliquots of 5.0 μM (0.1 mg/ml) of each protein were incubated for 20 min at different temperatures in PBS 1X buffer, centrifuged for 20 min at 16 000 × *g*, and 20 μl of supernatant were immediately loaded on 15% SDS-PAGE. The relative intensity of Comassie-stained *Ss*OGT bands were plotted as a function of temperature, considering as 100% the intensity of each protein band incubated at 25°C. Reported data are the mean ± SD of three independent experiments; (ii) Differential Scan Fluorimetry (adapting the protocol described in 22): each protein (25 μM; 0.5 mg/ml) was incubated in PBS 1X buffer and SYPRO Orange dye 1X, in a total volume of 30 μl. Samples were heated from 20 to 95°C in a Real-Time Light Cycler (Bio-Rad, Milan, Italy). Thermal stability scans were performed at 0.2°C/min (5 min/cycle with an increase of 1°C/cycle). Data were normalized to the maximum fluorescence value within each scan. Relative fluorescence intensities were plotted as a function of temperature; the obtained sigmoidal curve describes a two-state transition, where the *T*_m_ value represents the inflection point of the transition curve, as described by the Boltzmann Equation (2),

(2) \begin{equation*} y = {\rm LL} + \frac{{({\rm UL} - {\rm LL})}}{{1 + \exp \left( {\frac{{T_{\rm m} - x}}{a}} \right)}} \end{equation*}

where the values of minimum and maximum fluorescent intensities are LL and UL, respectively, and a represents the slope of the curve within *T*_m_ (22). Data are the mean ± SD of three independent experiments.

### Data analysis

Corrected data were fitted to appropriate equations by using GraFit 5.0 Data Analysis Software (Erithacus Software) or Prism Software Package (GraphPad Software) (23).

### Preparation of *Ss*OGT^m^

20 ml of 10 μM of the *Ss*OGT wt protein were incubated at 25°C for 24 h in the presence of *O^6^*-MG (10 mM, protein:inhibitor ratio 1:1000). To test the efficiency of methylation, an aliquot containing 50 pmols (1.0 μg) of *Ss*OGT from the methylation reaction was incubated with an equimolar amount of BG-VG at 70°C for 30 min and subsequently subjected to SDS-PAGE and fluorescence imaging: no fluorescent signal was detected, confirming the complete methylation of the protein. Unreacted inhibitor was removed using a HiTrap Desalting™ (GE, Healthcare) column pre-equilibrated in PBS 1X.

### Preparation of *Ss*OGT-C119A::dsDNA^m^ complex

To obtain the modified dsDNA molecule used in co-crystallization experiment (dsDNA^m^), the *O^6^*-methylguanine-containing oligonucleotide (5′-GCCATG[O^6^-MG]CTAGTA-3′, Primm, Milan, Italy) was annealed to the complementary oligonucleotide 5′-TACTAGCCATGGC-3′ (Eurofins MWG Operon). The resulting sample was mixed with the C119A protein solution (7 mg/ml in 20 mM phosphate buffer, pH 7.3 and 150 mM NaCl) at a protein:DNA molar ratio of 1:1.2, and incubated 1 h at room temperature before crystallization trials.

### Crystallization and data collection

Initial crystallization conditions for wild type, C119L and *Ss*OGT^m^ proteins and for the C119A::dsDNA^m^ complex were identified by means of a robot-assisted (Oryx4; Douglas Instruments), sitting-drop-based spare-matrix strategy using screen kits from Hampton Research and Qiagen. Wild-type *Ss*OGT crystals grew at 4°C in 4 μl drops obtained by mixing equal volumes of 9 mg/ml purified protein solution and precipitant 0.35 M potassium nitrate and 1.6 M ammonium sulfate in a final droplet volume of 1 μl. A single crystal was cryo-protected in precipitant solution containing 15% glycerol, mounted in a cryo-loop, and flash-frozen in liquid nitrogen at 100 K for further X-ray diffraction analysis. Single crystal diffracted at 1.85 Å of resolution at the ID23 synchrotron radiation (λ = 0.87 Å) (European Synchrotron Radiation Facility [ESRF], Grenoble, France). The diffraction data were indexed with *XDS* program (24), whose indexing score assigned crystal to the trigonal space-group R3 with the cell dimension a = 94.72 Å b = 94.72 Å c = 76.70 Å. Crystals of C119L mutant (8 mg/ml) were identified in the initial crystallization trials in the condition containing 4 M sodium formate as reservoir solution in a protein to reservoir ratio of 1:1, in a final droplet volume of 1 μl. Single crystal suitable for X-ray diffraction was manipulated as previously describe for wild-type protein and it diffracted at 2.6 Å of resolution at 100K at the ID23 beam line (λ = 1.89 Å) (ESRF, Grenoble, France). Indexing process with *XDS* program assigned the crystal to the cubic I432 space-group with the dimension a = b = c = 140.56 Å. *Ss*OGT^m^, obtained as described above, was concentrated to 7 mg/ml. Well-shaped single crystals grew in a 1 μl droplet containing equal volumes of protein and reservoir solution (0.1 M Bis-Tris pH 5.5, 0.1 M ammonium acetate, 17% w/v PEG 17000). Data collection was performed at BM30 syncroton radiation (λ = 0.979 Å) (ESRF, Grenoble, France), under cryogenic condition using 15% glycerol as cryo protectant in reservoir solution. The best crystal diffracted at 2.8 Å of resolution and it was assigned to the orthorhombic space group P212121 with the cell dimensions a = 48.49 Å b = 50.22 Å c = 142.02 Å, containing 2 molecule for asymmetric unit, with a Matthews parameter and a solvent fraction of 2.28 Å^3^ Da^−1^ and 45.98%, respectively. C119A::*O^6^*-MG-DNA single crystals grew in approximately 1 week in a 1 μl droplet of equal volume of protein-DNA complex solution and reservoir solution composed by 0.2 M Na/K tartrate 20% (w/v) PEG 3350. Diffraction experiments were conducted at 100 K using synchrotron radiation (λ = 0.972 Å) at the ID29 (ESRF, Grenoble, France). Data collection was performed up to 2.7 Å of resolution. Indexing score with *XDS* assigned crystal to the orthorhombic space group P212121 with the cell dimensions a = 41.76 Å b = 65.88 Å c = 97.52 Å. For all data sets described above, further data manipulations were carried out using *COMBAT* and *SCALA* from the CCP4 program suite (25). The data statistics of the solved structures are summarized in Table 1.

**Table 1.** Data collection, phasing, and refinement statistics

	SsOGT	SsOGT^m^	SsOGT-C119L	C119A::dsDNA^m^
Data collection				
Space Group	R3	P212121	I432	P212121
Unit cell (Å)	a = b = 94.72	a = 48.49	a = b = c = 140.56	a = 41.76
		b = 50.22		b = 65.88
	c = 76.70	c = 142.02		c = 97.52
	α = β = γ = 90°	α = β = γ = 90°	α = β = γ = 90°	α = β = γ = 90°
Wavelength (Å)	0.873	0.979	1.89	0.972
Resolution (Å)	1.8	2.8	2.6	2.7
Total reflections	83580	35418	78410	41645
Unique reflections	21897	8764	7600	7960
Mean(I)/sd(I)	11.4 (2.43) ^a^	10.1 (3.1) ^a^	25.6 (5.3) ^a^	10.62 (1.7) ^a^
Completeness (%)	99.9 (99.8) ^a^	97.5 (98.7) ^a^	99.9 (100) ^a^	98.5 (90.9) ^a^
Multiplicity	3.8 (3.7) ^a^	4.0 (4.1) ^a^	10.3 (10.3) ^a^	5.2 (5.2) ^a^
*R*_merge_ (%)	6.7	12.9	7.3	8.1
*R*_meas_ (%)	7.8	14.8	7.7	9.0

Refinement				
R_factor_/R_free_ (%)	16.0/18.2	19.0/29.0	17.5/24.2	21.3/27.1
Protein/DNA Atoms	1204	2380	1175	1707
Ligand atoms	24	-	-	-
Water molecules	211	12	45	6
R.m.s.d. bonds (Å)	0.007	0.010	0.009	0.012
R.m.s.d. angles (°)	0.98	1.27	1.31	1.29
Average B (Å^2^)				
Protein	25.3	17.9	35.3	63.8
Solvent	38.9	11.0	39.4	49.6

^a^Values in parentheses refer to the highest resolution shell.

### Structure determination, model building and refinement

The initial phases for wild-type *Ss*OGT, C119L mutant and *Ss*OGT^m^ structures were generated by molecular replacement with the program PHASER (26) of the PHENIX software suite (27) using *Sulfolobus tokodaii* OGT structure (PDB ID code:1WRJ) as the search model. The starting search model for C119A::*O^6^*-MeG-DNA complex structure consisted of *Sulfolobus tokodaii* OGT structure and the double stranded, methylated DNA molecule as crystallized in complex with hAGT (PDB ID code: 1T38) for the protein and DNA component, respectively. Initial model building was performed using AUTOBUILD of the PHENIX suite (28) followed by manual model building with the program COOT (29). Solvent molecules were added by ARP/wARP SOLVENT program from CCP4 program suite followed by structure refinement that was done with PHENIX (27). In the refined *Ss*OGT^m^ structure we noticed a large difference between *R*_work_ and *R*_free_ values. We deeply investigated all possible space groups suggested by the XDS analysis, and acceptable statistics could only be obtained by processing data in the space group P2_1_2_1_2_1_. Similarly an unambiguous solution of molecular replacement could only be found in P2_1_2_1_2_1_ space group. All figures illustrating structural analyses were generated with PyMol (http://www.pymol.org/) (30).

### Protein structure accession numbers

The atomic coordinates and structure factors of the *Ss*OGT wild type, C119L mutant, *Ss*OGT^m^ and C119A::dsDNA^m^ have been deposited in the Protein Data Bank (http://www.rcsb.org) under the accession codes 4ZYE, 4ZYH, 4ZYG, and 4ZYD, respectively

## RESULTS

### Structure-function analysis of free *Ss*OGT

**Figure 1.**
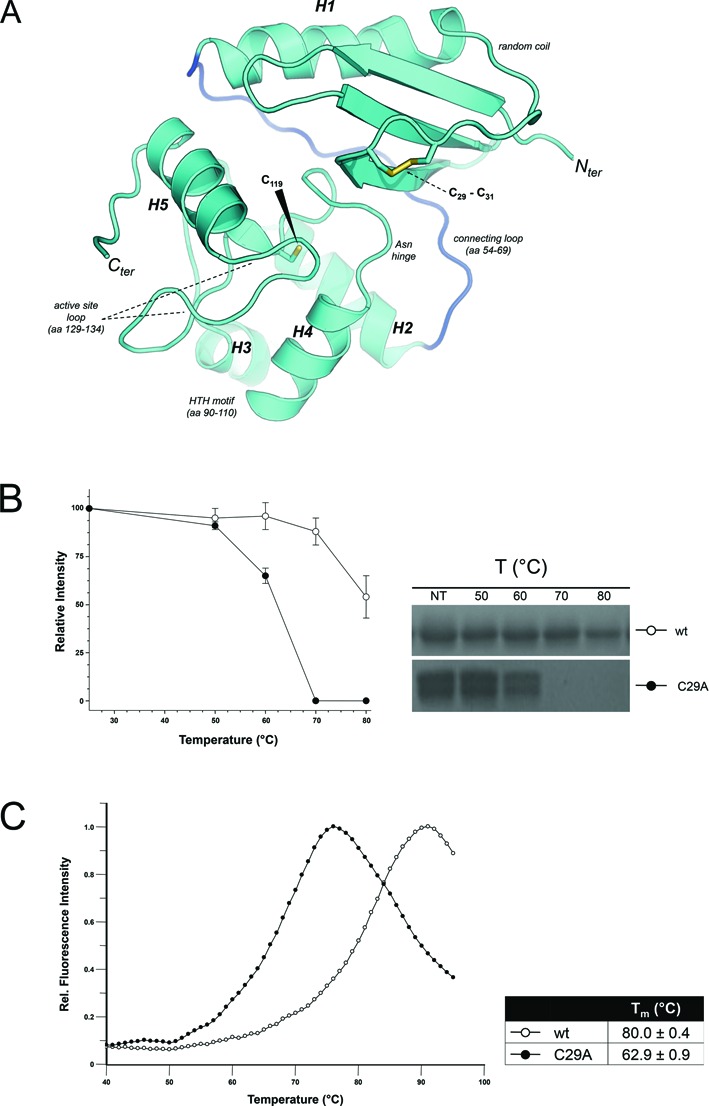
Structure–function of *Ss*OGT. (**A**) Cartoon representations of the crystal structure of wild-type ligand-free *Ss*OGT. Secondary structure elements and functional domains/motifs are indicated; residues commented through the text appears as sticks. (**B**) Thermal stability of the C29A mutant. The indicated proteins (0.1 mg/ml, 5 μM) were incubated for 20 min at the indicated temperatures; after incubation, samples were centrifuged for 20 min at 16 000 × *g* and supernatants were analyzed by SDS-PAGE (left); the relative intensity of the Coomassie-stained bands were plotted as a function of temperature (right), considering as 100% the reference value at 25°C for each protein; shown is the mean ± SD from three independent experiments. (**C**) Differential Scanning Fluorimetry (DSF). The relative fluorescence intensity values of each protein as a function of temperature were measured and used to obtain the *T*_m_ values. Data were obtained from three independent experiments.

The crystal structure of *Ss*OGT was solved at 1.8 Å resolution (Table 1). As observed for all AGTs structures present in the Protein Data Base (6–9,19,31–34), *Ss*OGT folds in two domains joined by a long connecting loop (a.a. 54–69) (Figure 1A). The N-terminal domain (a.a. 1–53) consists of an anti-parallel β-sheet, connected to a conserved α-helix (H1) by a random-coiled region, which is stabilized at its N-side by a disulphide bridge established between the C29 and C31. The C-terminal domain (a.a 70–151) houses the functional elements required for DNA binding and repair: (i) the catalytic C119 residue within the conserved PCHR signature; (ii) the helix-turn-helix motif (HTH), which binds the DNA minor groove and holds the arginine finger (R102) that participates to the modified base flipping out from the DNA base stacking; (iii) the ‘asparagine hinge’ that, together with the helix H4 of the HTH, defines one wall of the ligand-binding pocket; and (iv) the active site loop on the H4-facing side of the active site (Figure 1A). Previously, we demonstrated that mutation of the R102 residue reduces DNA binding efficiency, whereas mutation of five residues in the HTH motif abolishes the protein ability to form stable complexes with DNA, although both mutants are normally proficient in the trans-alkylation reaction (17).

An interesting feature, not previously reported for other AGTs, is the C29-C31 S-S bridge of the N-terminal domain. To test its role in the *Ss*OGT activity and stability, we produced the C29A mutant, which resulted as active as the wild-type protein in repair of a *O^6^*-MG-containing ds oligonucleotide at 50°C (Supplementary Figure S1B). In contrast, the C29A mutant was significantly less thermostable than the wild-type (Figure 1B): incubation at increasing temperatures showed that, whereas the wild-type protein remained 100% soluble after 20 min at 70°C, the C29A protein aggregated above 60°C. Quantitative analysis by Differential Scanning Fluorimetry (DSF) allowed calculation of a *T*_m_ of 80°C for *Ss*OGT and 60°C for C29A (Figure 1C). Thus, the S-S bond is not involved in DNA repair activity, but is an important structural element contributing to the impressive thermal stability of *Ss*OGT.

Alkyltransferase-like proteins (ATLs) share structural similarity with AGTs, but are catalytically inactive and are believed to act as DNA damage sensors (35). Although the general fold is shared by hAGT and *Schyzosaccharomyces pombe* ATL1, important differences were found in the catalytic loop and Asn hinge, resulting in larger size of the lesion-binding pocket in ATL1, which might account for its broad lesion recognition range (36). Superimposition of *Ss*OGT, ATL1 and hAGT structures showed the same differences, confirming the higher similarity of *Ss*OGT with hAGT as compared with ATL1 (Supplementary Figure S2).

### Structure-function analysis of dsDNA-bound *Ss*OGT

Different strategies have been adopted to trap the AGT-alkylated dsDNA complex and solve its structure: the wild-type hAGT has been crystallized crosslinked to oligonucleotides containing alkylated guanine analogues (8,9), and the hAGT C145S inactive mutant was co-crystallized with a more physiologic, *O^6^*-MG-containing, 13 base pair long oligonucleotide (dsDNA^m^) (8). These DNA-bound hAGT structures were found essentially superposable to each other and to ligand-free hAGT, suggesting that binding to DNA does not affect the protein overall structure (8,9).

**Figure 2.**
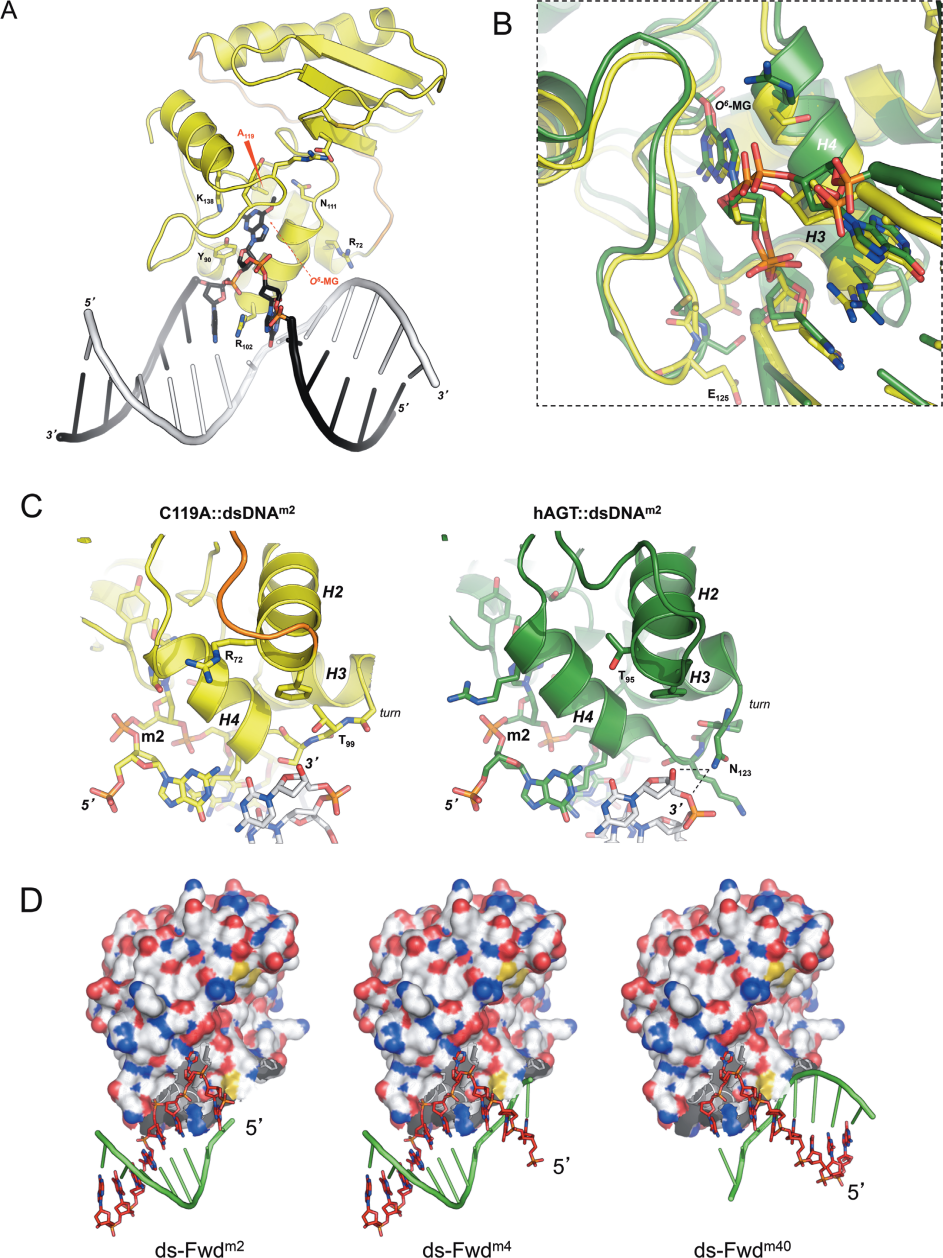
Structure-function of *Ss*OGT-DNA complex. (**A**) Cartoon representations of the crystal structure of the C119A mutant in complex with dsDNA^m^. (**B**) Detail of the interaction of superposed C119A (yellow) and hAGT (green) (PDB ID: 1T38) with the *O^6^*-MG ds DNA at the 3′ side of the lesion; (**C**) left, detail of the C119A::ds DNA^m2^ interactions at the 5′ side of the lesion; right, detail of the hAGT::ds DNA^m2^ interactions at the 5′ side of the lesion; in both images the damaged DNA strand is colored as the corresponding protein chain. (**D**) Structure-based models of C119A::ds oligonucleotides carrying the lesion in different positions (see Supplementary Table S1).

To solve the *Ss*OGT DNA-bound crystal structure, we obtained the C119A mutant, carrying a substitution of the catalytic C119 to prevent protein alkylation and subsequent dissociation from the repaired DNA substrate (data not shown). The crystal structure of the C119A::dsDNA^m^ (Figure 2A) was solved at 2.7 Å resolution (Table 1), revealing that a single *Ss*OGT monomer is able to occlude 4 base-pairs (bp) on dsDNA substrate, paralleling what observed for DNA-bound hAGT (37–39). The crystal structure of the ligand-free protein and of the C119A::dsDNA^m^ complex can be superposed with a 0.3 Å average root-mean-square deviation (r.m.s.d.) of Cα positions. Assuming that the structure of the C119A::dsDNA^m^ complex is similar to that of the physiologic complex formed by the wild-type protein, our analysis indicates that DNA binding does not substantially alter the protein architecture, as also shown for hAGT (8).

The C119A::dsDNA^m^ crystal structure was largely, but not completely, similar to that described for hAGT in complex with the same dsDNA^m^ substrate. The interaction network established by both proteins with the region of the modified strand at the 3′ side of the methylated base appears conserved. Indeed, in the C119A::dsDNA^m^ complex the phoshodiester bond between bases +1 and +2 downstream of *O^6^*-MG is clamped between the positive dipole momentum at the N-side of the H3 helix of the HTH domain and the backbone nitrogen of E125, at a mean 3.0 Å distance (Figure 2B). From this analysis we predict that at least 2 bases downstream to the *O^6^*-MG are needed to establish correct interactions, as shown for hAGT (40). In contrast, the protein-dsDNA interactions at the 5′ end of the *O^6^*-MG base appear less tight in the C119A::dsDNA^m^ structure compared to the human counterpart. Indeed, we found important differences in structure-based models of *Ss*OGT and hAGT in complex with a dsDNA^m2^, an oligonucleotide carrying the *O^6^*-MG lesion located one base from the 5′ end of the damaged strand (Supplementary Table S1). In the C119A::dsDNA^m2^ complex, the phosphodiester bond joining the last two bases at the 3′ end of the complementary intact strand is not engaged in contacts with the protein (Figure 2C, left panel), while it appears kept in place by the side chain of N123 in an equivalent model of the hAGT::dsDNA^m2^ complex (Figure 2C, right panel).

hAGT-catalyzed repair of dsDNA molecules containing a single *O^6^*-alkylguanine is directionally biased, as alkylated bases at positions near the 3′ end of the modified strand are repaired less efficiently than those located in the middle or at the 5′ end (40). We reasoned that, if this behavior reflects the protein-DNA complex architecture, *Ss*OGT might show a different bias toward the position of the lesion. To test this hypothesis we modified our previously developed competitive kinetic assay for *O^6^*-BG sensitive AGTs activity (17,19). Briefly, AGT proteins become covalently labeled when incubated with the fluorescent competitive inhibitor SNAP-Vista Green™ (hereafter BG-VG); the fluorescence intensity of the protein bands is a direct measure of the protein trans-alkylation activity. Moreover, the fluorescence intensity obtained in competition assays with BG-VG and unlabeled alkylated DNA is an indirect measure of the efficiency of DNA repair (17). This method was successfully applied to determination of kinetic constants for DNA trans-alkylation reaction and DNA repair activity by *Ss*OGT and *Mycobacterium tubercolosis* OGT (17,19). We have modified this assay to allow determination of DNA repair activity by *Ss*OGT by measuring the protein fluorescence intensity in competition assays performed in the presence of fixed amounts of BG-VG and increasing amounts of ds oligonucleotides containing a single *O^6^*-MG. This method allows rapid determination of an IC_50_, which can be converted to K_DNA_, giving an indirect measure of the efficiency of *O^6^*-MG repair by *Ss*OGT (Supplementary Figure S1A). The method was validated by determining the K_DNA_ of *Ss*OGT for the ds-UP^m^ oligonucleotide (Supplementary Table S1), which was comparable to the value previously reported (17) (Supplementary Figure S1A). We thus applied this method to determine the efficiency of repair by *Ss*OGT of 41 bp dsDNA substrates, each containing a single *O^6^*-MG at different positions along the molecule (Supplementary Table S1; see also Figure 2D). The efficiency of repair was comparable when the methylated base was located in the middle or within 3 bases from either the 5′ or the 3′ end. In contrast, the efficiency of repair was significantly reduced (about 6-fold) when the damaged base was located one base from the 5′ end, and dropped dramatically (more then 50-fold) when the lesion was located one base from the 3′ end (Table 2). Solution binding and AFM studies clearly indicated that hAGT binds both single- and double-stranded substrates in a cooperative fashion (38,39,41–44); the wild-type *Ss*OGT also showed cooperative binding when analyzed in EMSA assays with short oligonucleotides (17). The C119A protein showed similar behavior and bound all methylated oligonucleotides with comparable efficiency (Supplementary Figure S3), thus suggesting that the observed differences reflect the efficiency of *O^6^*-MG recognition and/or removal, rather than of unspecific DNA binding.

**Table 2.** Polarity of lesion recognition by *Ss*OGT

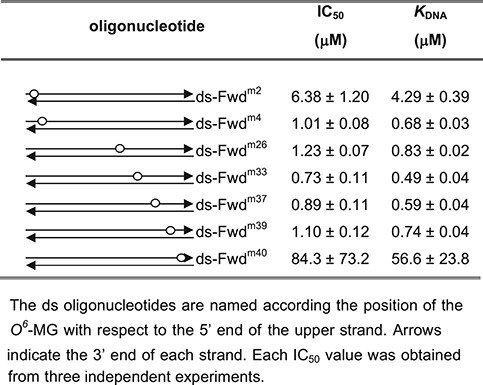		

These results show that *Ss*OGT, like hAGT, is highly inefficient in repairing lesions near the 3′ end of the molecule, in line with the conserved network of interactions formed with the DNA strand on the 3′ side of the lesion by both proteins (Figure 2B). In contrast, whereas two bases from the 5′ end of the molecule are sufficient for efficient repair by hAGT, *Ss*OGT requires at least 4 bases between *O^6^*-MG and the 5′ end for optimal activity (a situation illustrated by the central miniature in Figure 2D); in this latter case, positively charged residues of both helix H2 (e.g. R72 in Figure 2A) and helix H4 could efficiently contact the sugar-phosphate backbone of the complementary strand 3′ end, fully restoring the protein-DNA association potential.

### Effect of alkylation on *Ss*OGT stability

Methylated and benzylated forms of hAGT are highly unstable (37), and soaking of hAGT crystals in *O^6^*-alkyl-guanine substrates leads to crystals destabilization (7). Considering that *Ss*OGT is extremely stable at high temperature, but is also active at room temperature (17), we wondered whether it might be alkylated and remain relatively stable if kept at temperatures below its optimum. Consistently with this assumption, we obtained a homogeneously methylated protein form (*Ss*OGT^m^) by incubating purified *Ss*OGT with *O^6^*-MG at room temperature. Since the benzylated *Ss*OGT form (*Ss*OGT^b^) was not soluble, to test the effect of the presence of larger adducts in the protein active site, we constructed two site-directed mutants carrying substitutions of the catalytic C119 with an F or an L residue, mimicking a benzylated or isopropylated protein, respectively. Whereas the corresponding C145F and C145L mutants of hAGT were extremely unstable when expressed in *E. coli* cells (7), both *Ss*OGT mutants resulted stable in the same expression system and could be purified to homogeneity (data not shown).

**Figure 3.**
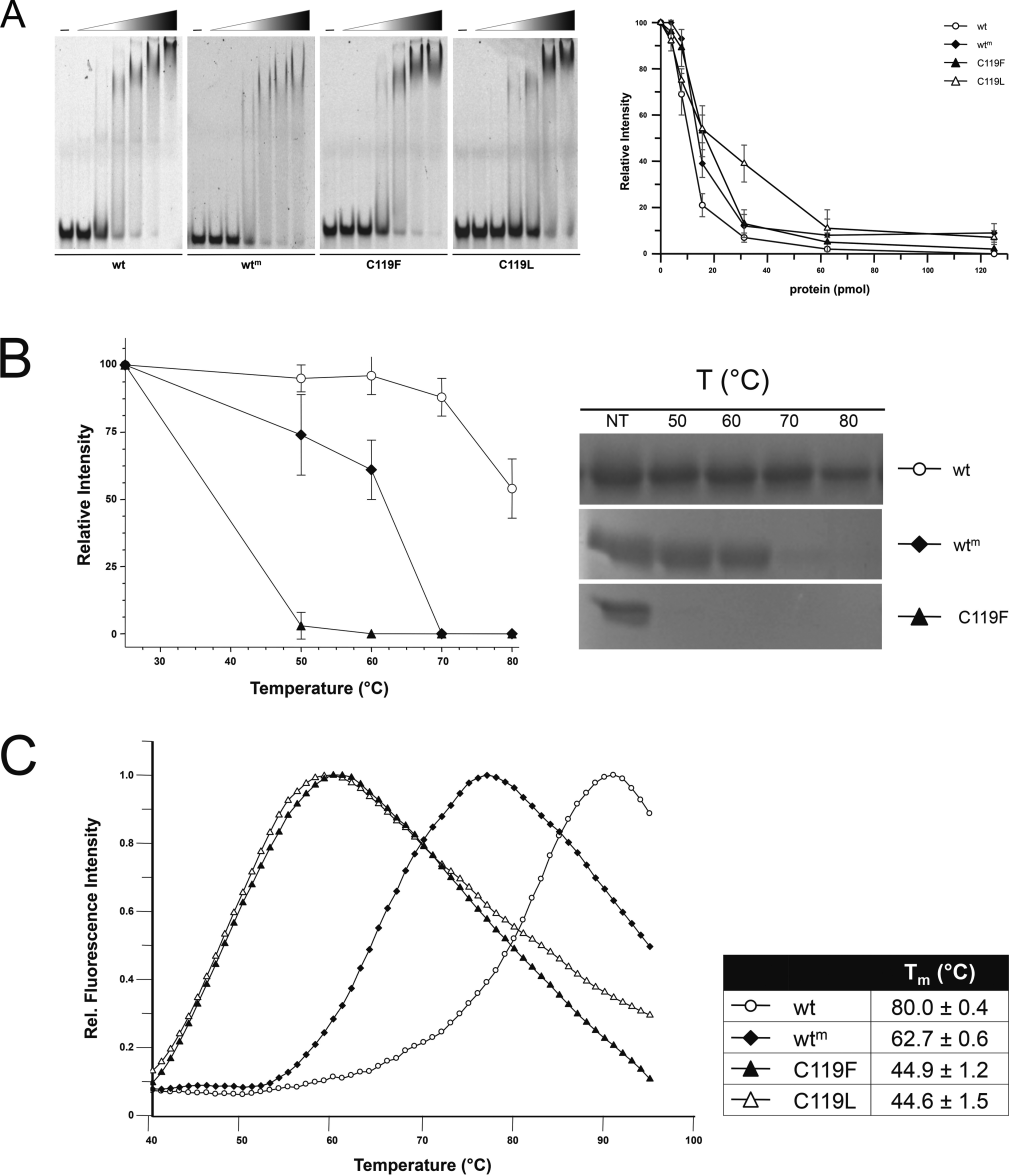
Effect of active site alkylation on *Ss*OGT activity and stability. **(A)** EMSA. Reactions (10.0 μl) containing increasing amounts of each indicated protein (0–25.0 μM) were incubated with the TAMRA-labeled ds A^+^/A^−^ oligonucleotide (0.2 μM; Supplementary Table S1) for 10 min at 37°C. Native polyacrylamide gels were analyzed by gel fluorescence imaging. The first lane of each gel is the no protein control. For quantification (right), the relative intensity of each band was plotted as a function of the protein concentration (pmol); data are from three independent experiments. **(B)** Thermal stability. Reactions were set and analyzed and quantified as described in the legend to Figure 1B. **(C)** DSF. Data were obtained from three independent experiments as described in the legend to Figure 1C.

When tested in EMSA analysis, *Ss*OGT^m^ and mutants were able to bind dsDNA, although with slightly reduced affinity as compared with the wild-type protein (Figure 3A), in line with results reported for hAGT, whose dsDNA binding affinity decreases to little extent when the protein is alkylated *in vitro* (37). We then analyzed the stability of the mutants and *Ss*OGT^m^ at different temperatures. Whereas, as shown above, the wild-type protein was 100% soluble after 20 min incubation at 70°C, complete aggregation was observed for *Ss*OGT^m^ at 70°C and for C119F at 50°C (Figure 3B). DSF analysis demonstrated the instability of these proteins, showing a *T*_m_ value of 60°C for *Ss*OGT^m^ and ca. 45°C for both the C119L and C119F mutants, compared with the wild-type protein (*T*_m_ = 80°C) (Figure 3C). Thus, the presence of alkylated groups bound to the catalytic C119 leads to *T*_m_ destabilization, whose extent is dependent on the size of the adduct in the active site.

### Effect of alkylation on *Ss*OGT structure

In order to elucidate the structural basis of the dramatic effect of alkylation on protein stability, we solved the crystal structure of the *Ss*OGT^m^ and *Ss*OGT-C119L proteins at 2.8 and 2.6 Å resolution respectively, whereas we failed to obtain suitable crystals of the C119F mutant, likely as a consequence of its intrinsic instability.

**Figure 4.**
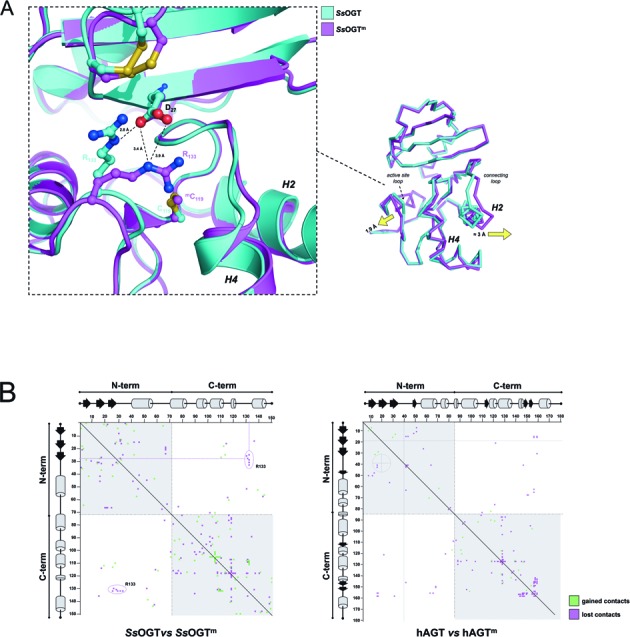
Comparative analysis of structures of free and methyalted *Ss*OGT. **(A)** Ribbon representation of the crystal structures of the two proteins upon optimal overlaying. Each protein chain is uniformly colored following the legend on the right of the picture. The arrows indicate the movements described in the text. An enlarged view of the D27 and R133 residues (drawn as sticks) appears in the inset on the left. **(B)** Distance difference matrix plot, obtained by the computation of Needleman-Wunsch sequence alignment by using the CMView v1.1.1 freeware, choosing amino acid side chain contact type and 8 Å cutoff. (left panel) methylated versus native *Ss*OGT; (right panel) methylated (PDB ID: 1EH7) versus native hAGT (PDB ID: 1EH6).

The analysis of the optimally superposed crystal structures of the ligand-free and *Ss*OGT^m^ proteins revealed conformational changes that occur in discrete regions of the molecule, upon the C119 methylation (Figure 4A). The only comparable structures available so far are those obtained by alkylating hAGT after crystallization (7); similarly to what observed in the human counterpart, in the *Ss*OGT^m^ structure we observed a 1.0 Å movement of the C-side of the recognition helix H4 and following Asn-hinge, a conformational change responsible for a modest increase of the ligand binding site size (Figure 4A). In addition, in the structure of *Ss*OGT^m^ the distance between the C-side of the conserved H2 helix and the H4 recognition helix is increased of approximately 3 Å with respect to the observed distance between the same structural elements in the ligand-free wild-type protein. Similarly, the distance between the active site loop and the H4 is increased of 1.9 Å in the *Ss*OGT^m^ structure. Alkylation of hAGT crystals induce shift of 0.5–1.5 Å Cα of the recognition helix away from the N-terminal domain (7).

An unbiased difference distance matrix plot of *Ss*OGT^m^ versus *Ss*OGT revealed that many interactions between aminoacid residues were lost or gained, suggesting overall reshaping of the molecule upon methylation (Figure 4B, left panel). Compared with analogous analysis performed on alkylated versus free hAGT (Figure 4B, right panel), many more interactions were affected upon *Ss*OGT methylation, thus suggesting that crystallization of the protein in its reacted form allow us to observe the repositioning of higher number of residues than that observed in the hAGT methylated in crystallized form. In both proteins most rearrangements occurred within the N- and C-terminal domain, respectively (Figure 4B, gray quarters) rather than between domains (white quarters); however, in *Ss*OGT^m^ a group of inter-domain interactions were lost in correspondence of the R133 residue (Figure 4B). Interestingly, in the *Ss*OGT structure, the R133 residue at the C-side of the active site loop of the C-terminal domain and the D27 residue at the C-side of the third β-strand of the N-terminal domain form an interaction at 2.6 Å distance (Figure 4A, inset). In the *Ss*OGT^m^ structure the distance between the carboxylic group of D27 and the guanidinium group of R133 is increased by 0.6 Å and the R133 side chain is rotated by ca 60° with respect to its position in the unmodified protein on the same plane, overall resulting in a 1.9 Å movement of the active site loop toward the solvent (Figure 4A). Distance increase from the D27 residue and rotation of the R133 residue was also observed in the superposition of the C119L structure with that of the wild type, although at lower extent (Supplementary Figure S4, inset), suggesting again that the modification of the D27–R133 interaction is a direct consequence of the active site modification. This observation prompted us to hypothesize that the C119 alkylation could have a role in the conformational change of the active site loop, which, in turn, might perturb the D27/R133 interaction, or alter the interaction geometry, ultimately triggering protein destabilization.

**Figure 5.**
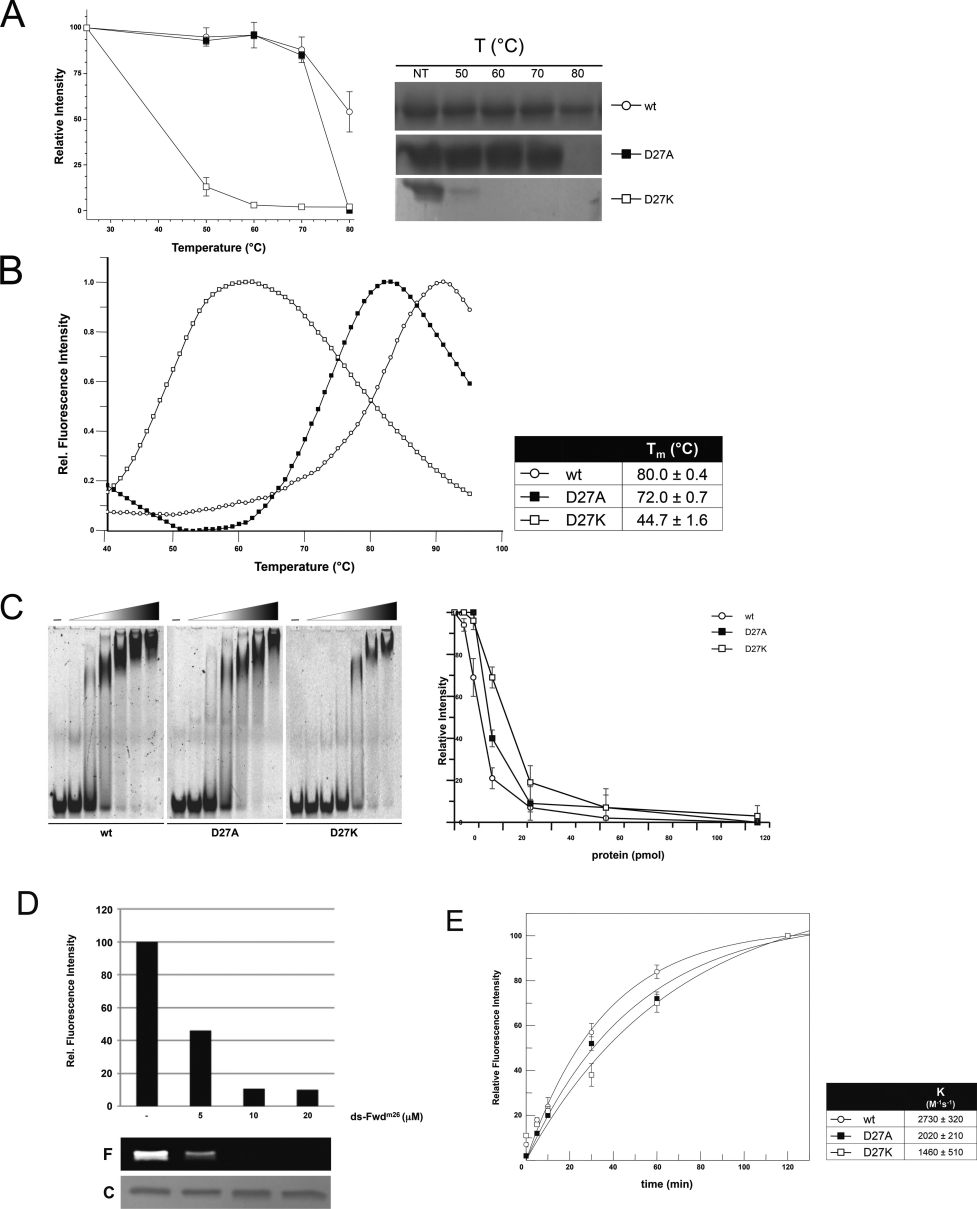
Effect of D27 residue mutations on *Ss*OGT stability and activity. **(A)** Thermal stability (see legend to Figure 1B; mean ± SD from three independent experiments). **(B)** DSF. (see legend to Figure 1C; data from three independent experiments). **(C)** EMSAs were performed and quantified as in the legend to Figure 3A. The first lane of each gel is the no protein control. **(D)** Direct DNA repair activity of *Ss*OGT D27K mutant. The protein (5 μM) was incubated for 6 h at 25°C in the presence of increasing amounts of the ds-Fwd^m26^ oligonucleotide, as indicated. Then, 10 μM of BG-VG was added and the incubation was prolonged for 2 h. The histogram reports the reduction of the fluorescence intensity of the protein band (shown in the gel labeled F) depending on the methylated oligonucleotide concentration in pre-incubation before BG-VG labeling. Equal amounts of protein were loaded in each lane, as shown by the Coomassie staining of the gel (C). **(E)** Trans-alkylation activity of D27 mutants. Second-order rate constants were obtained incubating the proteins at 25°C with BG-VG for increasing time spans; data were fitted as previously reported (17).

The active site expansion is not observed in the crystal structure of the C119L mutant, which rather shows a 1 Å movement of the H2 helix toward the Asn hinge (Supplementary Figure S4); therefore the C119L mutant is not as informative as the *Ss*OGT^m^ protein with respect to the description of structural rearrangements taking place upon trans-alkylation reaction.

### Alkylation-induced structural changes affecting the connection between the two domains impair *Ss*OGT stability and activity

In order to directly test whether the D27/R133 interaction plays a role in *Ss*OGT stability, we prepared two mutants carrying substitutions of the D27 residue, namely D27A and D27K, whose positive charge should eventually enhance the mutation destabilizing effect. In thermal stability assays, the D27A mutant was indeed destabilized with respect to the wild type, showing complete aggregation at 70°C; moreover, the D27K protein was dramatically less stable and aggregated completely above 50°C (Figure 5A); the *T*_m_ determined by DSF was of 72°C for D27A and 44.7°C for D27K, respectively (Figure 5B). Thus, these data confirmed our prediction that the interaction between D27 and R133 plays a crucial role in *Ss*OGT stability.

We then characterized the D27A and D27K activity. Both mutants were effective in dsDNA binding, although the D27K mutant showed slightly reduced binding efficiency as compared with the wild-type and the D27A proteins (Figure 5C). In addition, mutated proteins were tested in their DNA repair activity by using the fluorescent assay with *O^6^*-MG containing ds oligonucleotides described in Supplementary Figure S1. While the D27A mutant showed slight reduction of repair efficiency, as compared with the wild-type (IC_50_ of 2.26 ± 0.13 versus 1.23 ± 0.07 μM at 50°C), we could not calculate an IC_50_ value for the D27K protein at either 50 or 25°C, thus suggesting that the D27K protein is unable to repair DNA, or this activity is greatly impaired. A slow DNA repair activity by the D27K protein could be detected in prolonged (6 h) incubation at 25°C with methylated oligonucleotides (Figure 5D; note that under the same conditions the wild-type *Ss*OGT completes the reaction within few minutes; data not shown); thus, the D27K protein is not completely inactive, yet its DNA repair activity is strongly compromised. This result suggests that the disruption of the D27–R133 interaction not only affects the protein stability, but also its DNA repair activity even at low temperature, thus not as a consequence of thermal induced denaturation. To understand the reason of the D27 mutants DNA repair defect, we then determined the efficiency of the alkyl-transfer reaction independently of DNA binding and lesion recognition, using our previously developed assay based on the use of the synthetic substrate BG-VG (17). Interestingly, only marginal reduction of the catalytic efficiency of the covalent modification reaction at 25°C was observed for the D27K mutant, and no significant changes for the D27A, as compared with the wild-type protein (Figure 5E). Thus, the D27/R133 interaction does not play an important role in the alky-transfer reaction from free alkylated bases to the catalytic cysteine, while it greatly affects the efficiency of de-methylation of *O^6^*-MG bases in the context of dsDNA.

## DISCUSSION

*Ss*OGT proved to be a convenient model to unravel the structure-function relations of AGTs. Indeed, the overall architecture of the free and DNA-bound protein (this work), as well as details of its reaction mechanisms (17) are conserved between *Ss*OGT and mesophilic counterparts. Most important, the peculiar thermal stability of *Ss*OGT allowed us to obtain the protein in a post reaction form suitable to both biochemical analysis and crystallization, overcoming the restrictions to experimental manipulation imposed by the extreme instability of alkylated hAGT forms.

One peculiar feature of *Ss*OGT is the disulphide bond in its N-terminal domain; this structural element is not found in the corresponding domain of hAGT, which instead contains a zinc atom coordinated by two cysteine and two histidine residues (7). Previously, it was reported that nitric oxide synthase undergoes a conformational change with release of a Zn ion coordinated by four cysteines, coupled to formation of a disulphide bond involving two of such cysteines (45), thus suggesting that an exchange between Zn ion coordination and S-S bond is in principle possible. Although in the nitric oxide synthase this change appears to have regulatory function, it might occur in *Ss*OGT as an artifact due to Zn depletion during purification of the His-tagged protein through metal-chelating columns, inducing formation of the S-S bond. However, this is unlikely to be the case for two reasons: (i) the S-S bond is also present in the OGT protein from the strictly related *Sulfolobus tokodai* species, showing 68% aminoacid sequence identity with *Ss*OGT; although no functional data are available for this protein, its crystal structure (PDB entry 1WRJ) was obtained from the protein without any tag (http://www.ebi.ac.uk/thornton-srv/databases/cgi-bin/pdbsum/GetPage.pl?pdbcode=1wrj&template=header_records.html&r=getheader); (ii) the *Ss*OGT structure does not reveal any possible canonical tetrad for Zn coordination around the C29-C31 residues. Thus, the S-S bond appears to be a structural peculiarity of the thermophilic protein, and we showed that its disruption significantly destabilizes *Ss*OGT; it is possible that the N-terminal domain is involved in protein activity, as previously suggested (19,46), as well as in the overall stability of AGTs, although by means of different structural elements in different proteins.

The efficiency of DNA repair by *Ss*OGT is highly dependent on the *O^6^*-MG position, and its optimal activity requires at least three bases at both sides of the lesion, consistent with the contacts between the protein and both DNA strands in crystal structure. Interestingly, whereas contacts formed with the DNA strand at the 3′ side of the lesion are important for both hAGT and *Ss*OGT, those formed with bases at the 5′ side seem more important for the latter. One possible explanation for this difference is that *Ss*OGT needs to form stronger contacts at both sides of the DNA substrate to assist stabilization of the DNA-protein complex at higher temperatures, and/or hold the bound DNA in a correct ds conformation, counteracting thermal denaturation and facilitating lesion removal.

Alkylation-induced instability of hAGTs is a well known process whose molecular mechanism is, however, poorly understood, mainly due to the instability of alkylated hAGT and its C145F and C145L mutants (7,37). Thanks to the relative stability of *Ss*OGT^m^, and of C119L and C119F mutants at mild temperatures, we could obtain direct and quantitative data on the protein stability in correlation with the active site status, as well as insights in the structural modifications occurring upon methylation in solution, thus in conditions more physiological than those in which the structures of alkylated hATG could be obtained (7). Indeed, although structural rearrangements were observed upon alkylation of hAGT crystals, Daniels and coworkers predicted that such rearrangements would be even larger in solution (7). Consistently, we found extensive remodeling of interactions between aminoacid residues upon methylation and larger movements in the backbone structure of *Ss*OGT^m^, as compared with alkylated hAGT. These data support the correlation between active site alkylation, conformational changes and protein unfolding (7). Our data are also in line with the observation that alkylated *Ss*OGT undergoes degradation after treatment of *S. solfataricus* cells with alkylating agents (17): at the physiological growth temperature (75–80°C) alkylated *Ss*OGT is destabilized, which might target the protein to degradation pathways, either directly or after some still unidentified post-translational modification.

**Figure 6.**
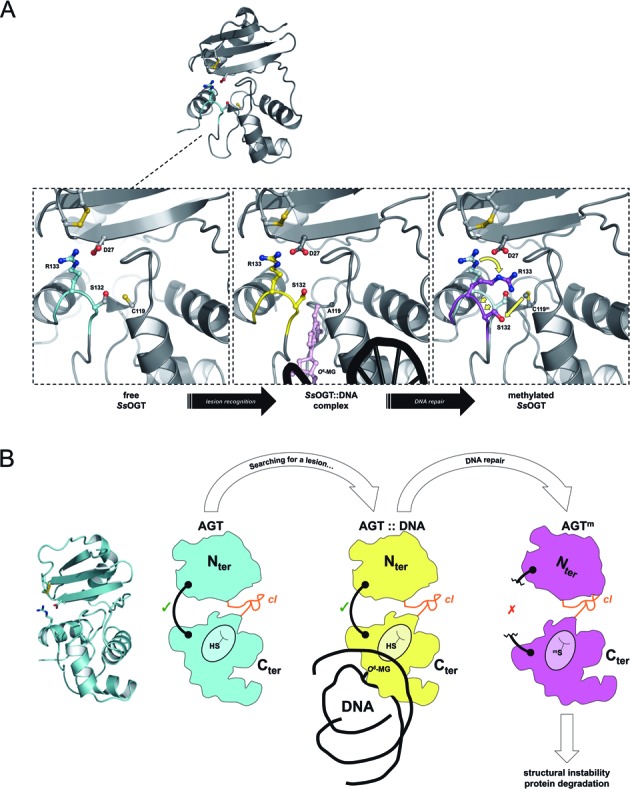
**(A)** Details of the D27–R133 interaction during the reaction cycle and its methylation-induced modification. **(B)** Model of the conformation modification induced by alkylation-induced perturbation of inter-domain interactions.

Our structural and biochemical data show that the D27 residue of the N-terminal domain plays an important role in both *Ss*OGT activity and stability, through the formation of an interaction with the R133 residue of the catalytic C-terminal domain (Figure 6A). Intriguingly, the remarkable extent of D27K destabilization is strikingly similar to that of the C119F and C119L mutants, showing that the same effect on protein stability is obtained by acting on two completely different residues. Moreover, the D27/R133 interaction is important not only to maintain *Ss*OGT folding at high temperature, but also to allow its activity even at low temperature. Exploiting our different assays, which allow dissection of the *Ss*OGT reaction, we showed that the D27–R133 interaction is not involved in the trans-alkylation reaction *per se*, whereas it is required for the protein to repair efficiently the alkylated base in the DNA context. An attractive hypothesis is that C119 alkylation-induced perturbation of the D27–R133 interaction weakens the contacts between the two domains, impairing optimal co-ordination between the N- and C-terminal protein domains, which in turn might be crucial both for DNA repair and maintenance of the correct protein folding.

We could also obtain the first crystal structure of an AGT mutant carrying a substitution of the catalytic cysteine with a leucine, mimicking an isopropylic group; the hAGT corresponding mutant was extremely unstable (7). The crystal structure of the C119L protein showed that the conformational modifications occurring in this mutant at the level of the D27–R133 interaction recall those occurring in the methylated protein. However, it is also important to note that not all the structural modifications observed in *Ss*OGT^m^ are also present in the C119L protein, thus suggesting that the bulky adduct in the active site inserted by mutation may not completely reproduce the effect of the conformational changes triggered by alkylation. Indeed, alkylation takes place in the correctly folded protein, whereas the mutant accommodates the large adduct during its folding, which might affect the protein conformation in a different manner. These results suggest that structural and functional data obtained with substitution mutants should be carefully considered.

The inter-domain D27–R133 interaction is not conserved in the structures of other AGTs, as a consequences of poor sequence conservation at level of the N-terminal domain of AGTs from different species. However, in the case of *Thermococcus kodakaraensis Tk*-MGMT the two domains are connected by an ion-pair network formed by the R50–E93–R132 residues. Although no data are available on the effect of alkylation on this interaction, mutation to alanine of the E93 residue, located at the center of the network, destabilized the protein (32,47). In hAGT, alkylation causes disruption of the active site hydrogen-bond network and perturbs the hydrophobic packing between the N-hinge and the N-terminal domain, which gives an important contribution to the interface between the two protein domains (7). Upon alkylation, movements of the HTH helix H6 and collision of the N137 residue with the alkyl adduct disrupt three H-bonds formed by the N137 residue. Most important, mutation of N137 to alanine resulted in dramatic hAGT destabilization (48). Thus, although in three AGTs the connection between the two domains are provided by different structural elements, these observation suggest a common theme for alkylation-induced destabilization through perturbation of the connection between the two domains, triggering protein destabilization (Figure 6B).

## Supplementary Material

SUPPLEMENTARY DATA
