# Supplementary material for: Structure-function relationships governing activity and stability of a DNA alkylation damage repair thermostable protein
Source: Nucleic Acids Res. 2015 Oct 10;43(18):8801–16. doi: 10.1093/nar/gkv774 (PMC4605297; doi:10.1093/nar/gkv774)
Supplement: SUPPLEMENTARY DATA [file supp_43_18_8801__index.html]

Structure-function relationships governing activity and stability of a DNA alkylation damage repair thermostable protein — Structure-function relationships governing activity and stability of a DNA alkylation damage repair thermostable protein — SUPPLEMENTARY DATA 

# Structure-function relationships governing activity and stability of a DNA alkylation damage repair thermostable protein

## SUPPLEMENTARY DATA

- SUPPLEMENTARY DATA
