# Supplementary material for: Structure-function relationships governing activity and stability of a DNA alkylation damage repair thermostable protein
Source: Nucleic Acids Res. 2015 Oct 10;43(18):8801–16. doi: 10.1093/nar/gkv774 (PMC4605297; doi:10.1093/nar/gkv774)
Supplement: SUPPLEMENTARY DATA [file supp_gkv774_nar-01491-h-2015-File021.pdf]

Supplementary Table 1: *List of the oligonucleotides used in this work*. The bold M represents the damaged *O*^{6}-methyl-guanine.

name	sequence	note
**D27A^{mut}**	5′-ataagggatttattatgttggctttctgtgactgtgtgg-3′	D27A mut underlined
**D27K^{mut}**	5′-ataagggatttattatgttgaaattctgtgactgtgtgg-3′	D27K mut underlined
**D27^{rev}**	5′-caacataataaatcccttatcgtccttagcaacag-3′	-
**C29A^{mut}**	5′-gatttattatgttggatttcgctgactgtgtggaagg-3′	C29A mut underlined
**C29A^{rev}**	5′-gaaatccaacataataaatcccttatcgtccttagc-3′	-
**C119A^{mut}**	5′-cccattcttttaatcataccagctcaccgagtaatagc-3′	C119A mut underlined
**C119L^{mut}**	5′-cccattcttttaatcataccactgcaccgagtaatagc-3′	C119L mut underlined
**C119F^{mut}**	5′-cccattcttttaatcataccattccaccgagtaatagc-3′	C119F mut underlined
**C119^{rev}**	5′-tggtatgattaaaagaatgggattttagataaagcc-3′	-
**UP^{m}**	5′-ggacactgtacgttaaggcg**M**atcgaattaggattaa-3′	Ref. 17
**DOWN**	5′-ggttaatcctaattcgatcgccttaacgtacagtgt-3′	Ref. 17
**Fwd**	5′-ggcagtaggcctagcatgacaatctgcattggtgatcacgg-3′	-
**Fwd^{m40}**	5′-ggcagtaggcctagcatgacaatctgcattggtgatcac**M**g-3′	-
**Fwd^{m39}**	5′-ggcagtaggcctagcatgacaatctgcattgtggatga**M**cg-3′	-
**Fwd^{m37}**	5′-ggcagtaggcctagcatgacaatctgcattgtggat**M**agcg-3′	-
**Fwd^{m33}**	5′-ggcagtaggcctagcatgacaatctgcattgt**M**gatgagcg-3′	-
**Fwd^{m26}**	5′-ggcagtaggcctagcatgacaatct**M**cattggtgatcacgg-3′	-
**Fwd^{m4}**	5′-ggc**M**gtaggcctagcatgacaatctgcattggtgatcacgg-3′	-
**Fwd^{m2}**	5′-g**M**cagtaggcctagcatgacaatctgcattggtgatcacgg-3′	-
**Rev**	5′-cgctcatccacaatgcagattgtcatgctaggcctactgcc-3′	-
**Rev^{4}**	5′-ccgtgatcaccaatgcagattgtcatgctaggcctaccgcc-3′	-
**oligo A^{+}**	5′-*gcaacaacgttgcgcaaactattaactggcgaactactta-3′	*= TMR-labelled (Ref.17)
**oligo D^{-}**	5′-taagtagttcgccagttaatagtttgcgcaacgttgttgc-3′	Ref. 17
**O^{6}-MeG-DNA^{+}**	5′-gccatg**M**ctagta-3′	
**O^{6}-MeG-DNA^{-}**	5′-tactagccatggc-3′	

Supplementary Figure 1. Principle of the modified DNA repair activity assay and its application to *Ss*OGT and C29A mutant. (A) The curve was obtained by fitting the relative fluorescence intensity (RFI) of each protein band obtained after incubation of fixed equimolar concentrations of *Ss*OGT and SNAP-Vista Green™ (BG-VG; 5 μM) with increasing concentrations of the ds-UP^{m} oligonucleotide (Supplementary Table 1), as indicated. Incubation was for 10 min at 50 °C under standard conditions (17). The lower panel shows a typical SDS-PAGE used to calculate the data above; each lane contains 2.0 μg of *Ss*OGT. The gel was scanned for fluorescence (F) and was stained with Coomassie blue (C); the relative fluorescence intensity values were obtained by correcting the absolute fluorescence of each band for its intensity in the Coomassie-stained image. The IC_{50} value was obtained from three independent experiments. *Ss*OGT wild type K_{DNA} previously determined for the ds-UP^{m} DNA (17) is comparable to that determined with the current method. (**B**) IC_{50} values for wild type *Ss*OGT and the C29A mutant were calculated following the method described in A, using the oligonucleotide ds-Fwd^{m26} (Supplementary Table 1).

Supplementary Figure 2. Comparative analysis of structures of free *Ss*OGT, hAGT and ATL1. Ribbon representation of the crystal structures upon optimal overlaying. Each protein chain is uniformly coloured following the legend on the right of the picture. An enlarged view of the active site loop and the Asn-hinge appears in the inset on the left.

Supplementary Figure 3. Binding of *Ss*OGT-C119A to oligonucleotides containing an *O*^{6}-MG at different positions. (A) Reactions (10.0 μL) containing increasing amounts of protein (0-25.0 μM) were incubated with of the appropriate oligonucleotide (0.2 μM) for 10 min at 37 °C. M indicates the position of the *O*^{6}-MG with respect to the oligonucleotide 5' end. Reactions were loaded on native agarose gels which were stained by ethidium bromide and analyzed by gel fluorescence imaging. The first lane of each gel is the no protein control. **(B)** The relative fluorescence intensity (RFI) was plotted as a function of the protein concentration (pmol); data are from three independent experiments.

Supplementary Figure 4: Comparative analysis of structures of free SsOGT and the C119L mutant. Ribbon representation of the crystal structures of SsOGT and C119L upon optimal overlaying. Each protein chain is uniformly colored following the legend on the right of the picture. The arrow indicates the movements described in the text. An enlarged view of the D27 and R133 residues (drawn as sticks) appears in the inset on the left.

Suppl Fig. 1

A

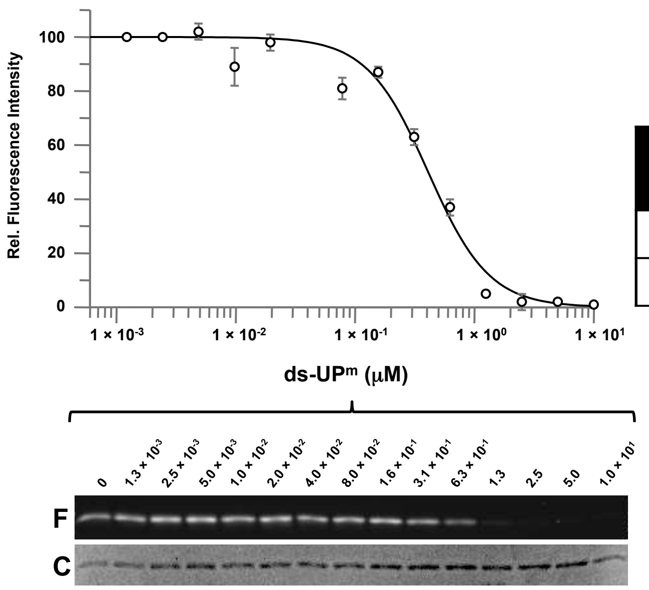

IC_{50} (μM)	K_{DNA} (μM)	notes
-	0.77 ± 0.01	from ref. 17
0.41 ± 0.04	0.28 ± 0.03	this study

B

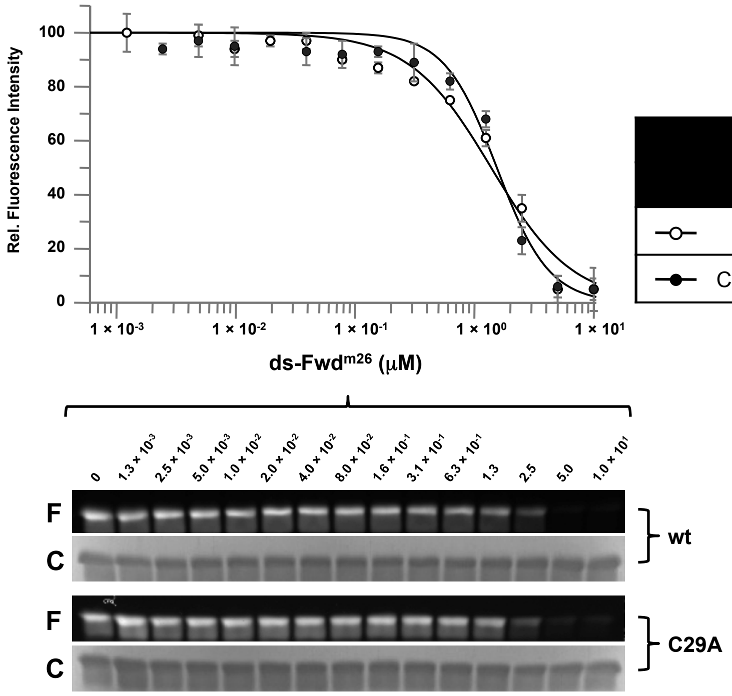

	IC_{50} (μM)	K_{DNA} (μM)	notes
○ wt	1.23 ± 0.07	0.83 ± 0.02	this study
● C29A	1.54 ± 0.13	1.03 ± 0.09	this study

Suppl Fig. 2

A

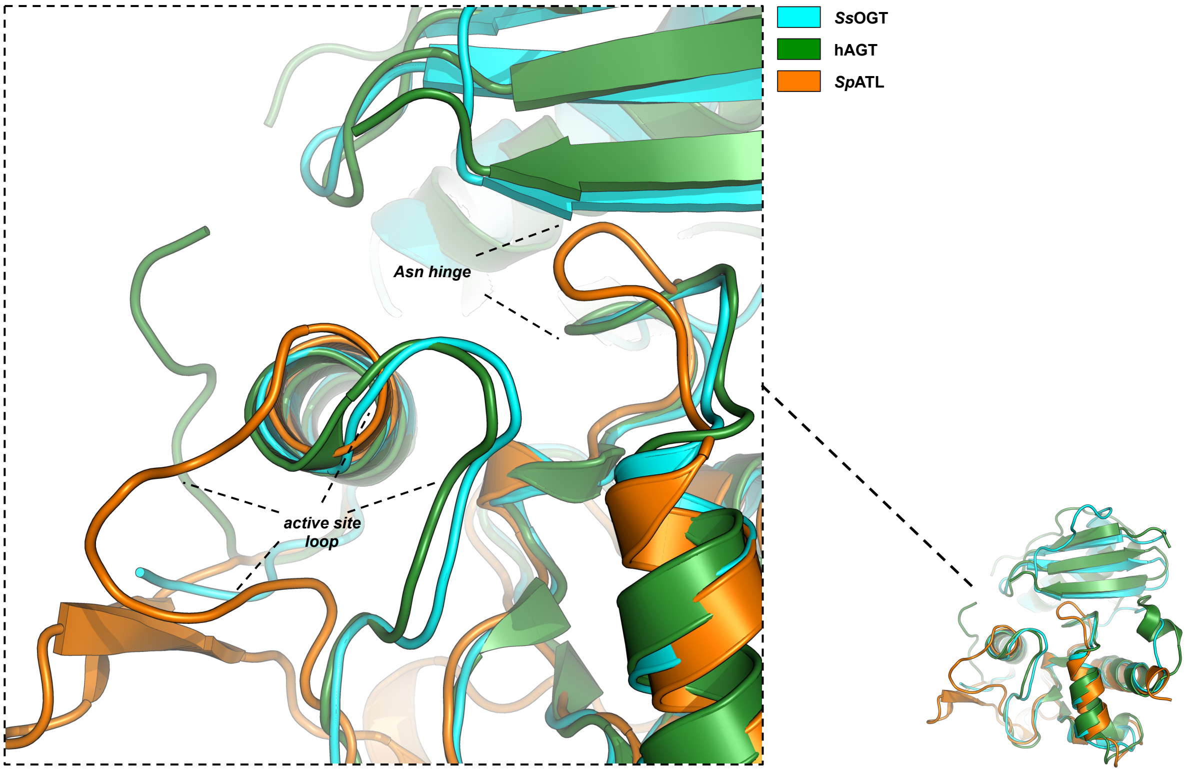

Suppl Fig. 3

A

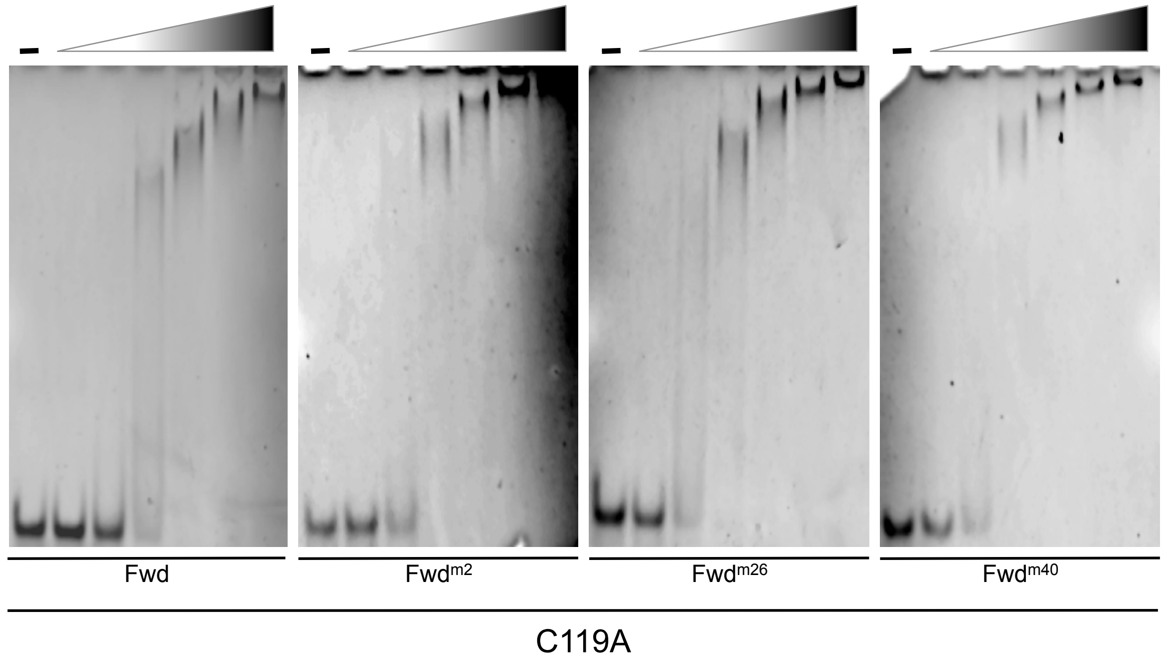

B

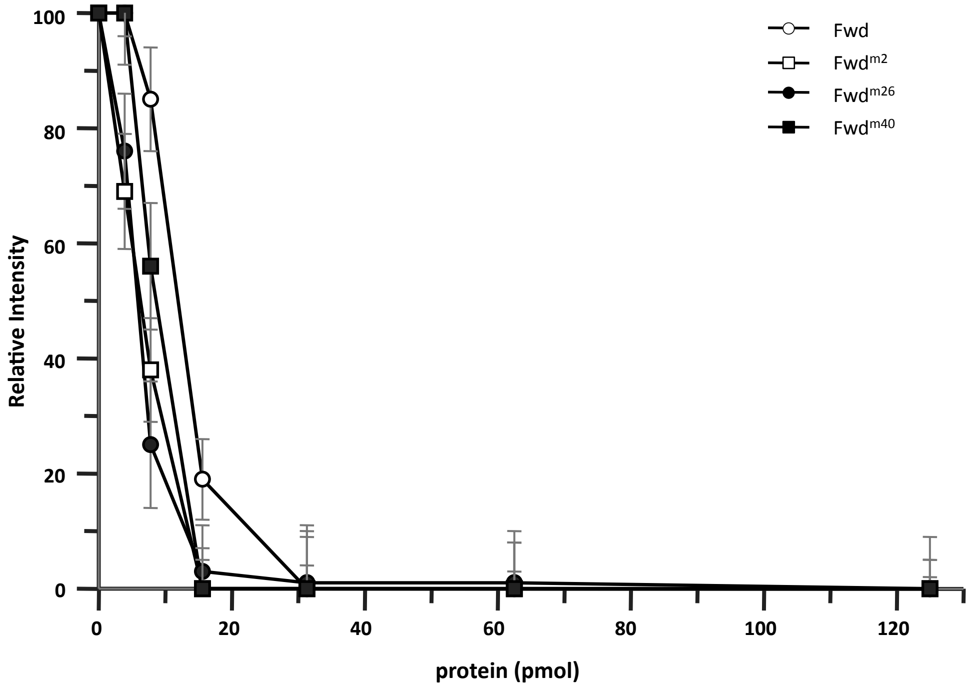

Suppl Fig. 4

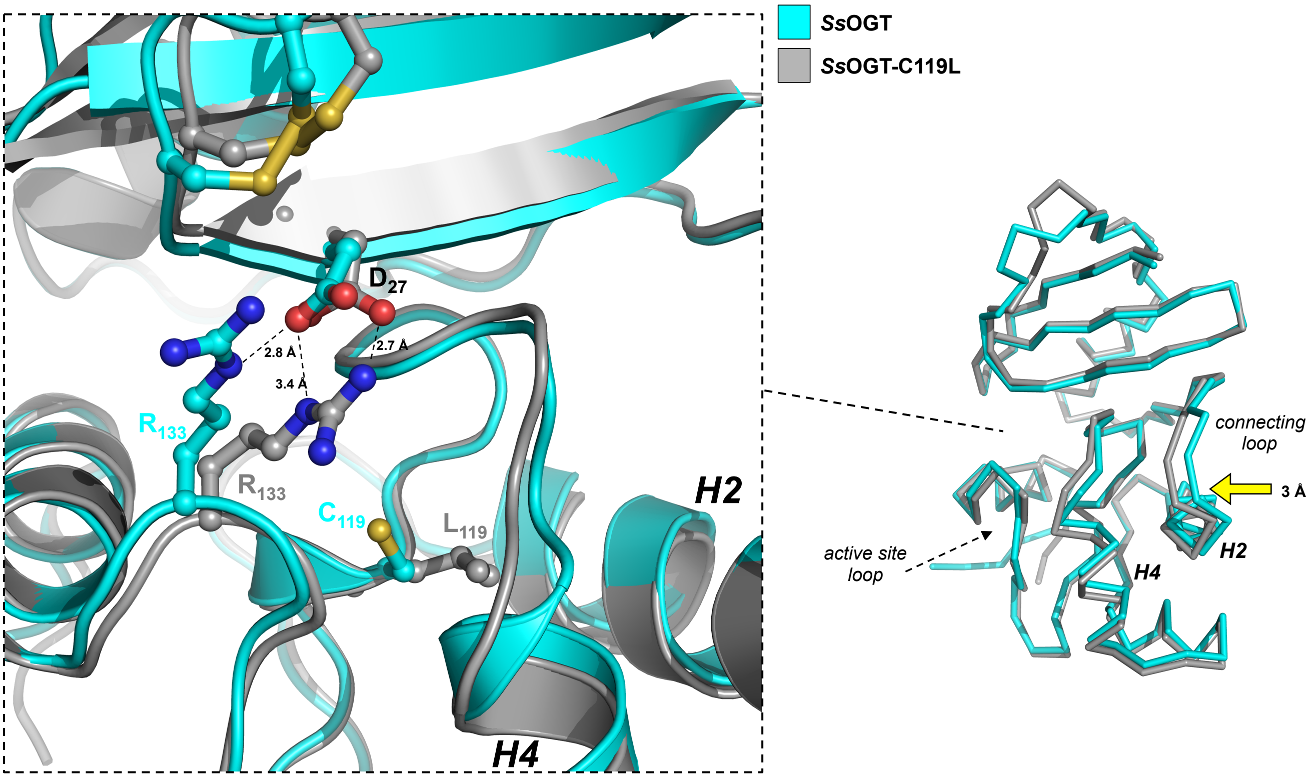

SsOGT
SsOGT-C119L
D27
2.8 Å
3.4 Å
2.7 Å
R133
R133
C119
L119
H2
H4
connecting loop
3 Å
active site loop
H2
H4
